# The impact of the Maritime Labor Convention on seafarers’ working and living conditions: an analysis of port state control statistics

**DOI:** 10.1186/s12889-020-09682-6

**Published:** 2020-10-21

**Authors:** Marina Liselotte Fotteler, Despena Andrioti Bygvraa, Olaf Chresten Jensen

**Affiliations:** 1 DigiHealth Institute, Neu-Ulm University of Applied Sciences, Neu-Ulm, Germany; 2 School of Medicine, Ulm University, Ulm, Germany; 3 Center for Maritime Health and Society, Institute of Public Health, University of Southern Denmark, Niels Bohrs vej 9, 6700 Esbjerg, Denmark

**Keywords:** Maritime labour convention, MLC2006, Port state control, Memoranda of understanding, Seafaring, Working conditions

## Abstract

**Background:**

The Maritime Labour Convention, 2006 (MLC2006) entered into force in August 2013 and is a milestone for better working and living conditions (WLC) for seafarers. As of March 2020, 96 countries have ratified the MLC2006, covering more than 90% of the world’s shipping fleet. A system of port state control (PSC) allows ratifying countries to inspect any foreign ship arriving in their ports for compliance with the convention. It is intended as a second safety measure for the identification of substandard ships that sail all over the world. Nine regional agreements, so-called Memoranda of Understanding (MoU), have been signed to coordinate and standardize PSC inspections and to increase efficiency by sharing inspections and information. This paper uses public PSC statistics to evaluate the impact of the MLC2006.

**Methods:**

A preliminary analysis using registered tonnage and MLC2006 ratification was conducted and seven MoU were selected for the analysis. The annual reports of these MoU have been viewed in September 2019. Numbers on annual inspections, deficiencies and detentions and in particular data for deficiencies related to living and working conditions and certificates and documents, have been extracted and analyzed for the years 2010 to 2017.

**Results:**

Across the eight-year period analyzed, inspection numbers remained stable among all MoU authorities. Deficiencies overall and deficiencies related to WLC declined, indicating an improvement in conditions overall and an increased focus on seafarers’ conditions on board. After the MLC2006 entered into force, three MoU reported WLC-ratios above 14%, while the numbers didn’t rise above 10% in the other four authorities. Deficiencies related to certificates and documents did not rise significantly between 2010 and 2017. Two European MoU showed the highest ratios for deficiencies in both categories analyzed.

**Conclusion:**

The analysis confirmed that an increasing attention is being paid to the inspection of working and living conditions, especially in European countries. However, a clear positive impact of the MLC2006 could not be determined from the PSC statistics in this analysis. A large variation still exists among the MoU, a fact that demands increased efforts for harmonization of PSC procedures.

**Supplementary information:**

**Supplementary information** accompanies this paper at 10.1186/s12889-020-09682-6.

## Background

Seafarers have historically been exposed to hazardous conditions that can negatively impact physical and mental health. They often face poor diet and accommodation, shift work, exploitation and financial pressure through non-compliance with contracts or non-payment of wages [1–5]. The Maritime Labour Convention, 2006 (MLC2006) is a milestone for the improvement of seafarer health and wellbeing. Adopted by the International Labour Organization (ILO) in 2006 it entered into force in August, 2013. In five chapters, the MLC2006 covers (i) the minimum requirements for seafarers to work on a ship, (ii) the conditions of employment, (iii) accommodation, recreational facilities, food and catering, (iv) health protection, medical care, welfare and social security protection and (v) compliance and enforcement [6]. The MLC2006 is continually evolving and regular amendments ensure the future validity and relevance of the convention. As of March 2020, 96 countries have ratified the MLC2006, covering more than 90% of the world’s shipping fleet [7].

Once a nation has ratified the Convention, the regulations become part of national law and thus binding for ships under its flag. The primary responsibility for ensuring compliance rests with the ratifying country (flag state control). The MLC2006 also requests that ships, especially those of non-ratifying countries, *“do not receive more favourable treatment”* (Article V, 7). The MLC2006 and many other conventions such as the International Convention for the Safety of Life at Sea [8] achieve this fair competition by a system of port state control (PSC). PSC allows ratifying countries to inspect foreign ships arriving in their national ports to ensure that international regulations and requirements are adhered to. It creates an additional incentive for ship owners to make sure their ships comply with existing standards. PSC has been a part of maritime law since the early twentieth century and is intended as a second safety measure for the identification of substandard ships [9–11]. Since the 1980s, several regional agreements, so-called Memoranda of Understanding (MoU), have been signed to coordinate and standardize PSC inspections and to increase efficiency by sharing inspections and information. Starting with the Paris MoU established by 26 European maritime states and Canada in 1982, eight other MoUs followed, now including all the world’s main ports [10]. The other MoU are the Tokyo MoU, Indian Ocean MoU, Caribbean MoU, Abuja MoU, Acuerdo Latino (Acuerdo Viña del Mar), Mediterranean MoU, Black Sea MoU and Riyadh MoU. The United States (US) conduct their own PSC via the US Coast Guard [12].

Port state inspections for all international conventions are conducted by a port state control officer (PSCO) and regulated by a set of guidelines. In case of the MLC2006, the PSCO starts by checking the Maritime Labour Certificate and the Declaration of Maritime Labour Compliance on ships flying the flag of ratifying countries. Both documents count as evidence for compliance with the MLC2006. More detailed inspections are carried out in case of document deficiencies, seafarer complaints or other suspicions. Ships sailing under flags of non-ratifying countries are also suspect to more thorough inspections [13]. The PSCO records detected deficiencies for different categories and can impose consequences on the ship depending on the severity of the violations. These are to demand (i) an immediate rectification prior to the planned departure, (ii) a rectification in the next port, (iii) a rectification within 14 days or (iv) the detention of the ship until all deficiencies are rectified. Detentions are only imposed if the deficiencies are hazardous to safety, health or the environment [9, 14]. Even though all MoU agreements are based on the original text written for the Paris MoU and therefore have similar legal structures, differences exist regarding inspection procedures, targeting criteria and institutional agreements [12] (additional information in supplementary file 1).

The MLC2006 celebrated its five-year anniversary in August 2018 and it is essential to determine whether the Convention achieved its goal of improving seafarers’ health and wellbeing. Few studies are available on the impact of the MLC2006. In a reference analysis, Noufal et al. [15] came to the conclusion that Egyptian law is inferior to the regulations enforced by the MLC2006 and urge their government to ratify the convention. Results from other studies however paint an unclear picture and suggest that the MLC2006 might not have entirely brought around the expected changes. Some authors suggested that the impact did not reach the anticipated level due to implementation difficulties by the different ratifying flag states [16]. Others claim the MLC2006 doesn’t go far enough and must be revised [17, 18]. A recent pilot study came to the conclusion that the MLC2006 radically increased paperwork for many seafarers while failing to adequately address many of the most pressing issues including manning, work and rest hours, food quality and recreational facilities [19]. The Mission to Seafarers publishes quarterly reports on the results of the Seafarers’ Happiness Index, which frequently mention the MLC2006 and remaining issues related to its provisions. Currently, the biggest problems are low manning, rest hours and the difficulty to adhere to them, low food quality, lack of recreational facilities, non-payment and a ban of shore leave opportunities – all issues addressed in the text of the MLC2006 [20].

This paper takes the approach to analyze the implementation of the MLC2006 from the perspective of PSC statistics. PSC is widely recognized as one of the most important measures for safety at sea, some even deem it more effective than flag state control [9, 16]. Statistical analyses on the effectiveness of PSC however are still lacking [9]. In this paper, data reported between 2010 and 2017 by seven of the world’s nine MoU were used to evaluate the impact of the MLC2006. It has been stated that the inspection of working and living conditions (WLC) on board did not receive a lot of attention during PSC procedures prior to the MLC2006 [17]. It was hypothesized that deficiencies related to WLC increased after the MLC2006 entered into force and decreased again after some years due to improved conditions. Other studies have used PSC data to investigate the impact and the effectiveness of PSC [9, 11, 13, 16, 21–26]. Some of these studies also focus on labor conditions and MLC2006-related inspection results. Most available research, analyzes numbers from only one MoU for one or two years, mainly the two most active PSC organizations, the Paris MoU and the Tokyo MoU. Two other studies that attempted a comparison of various MoU data over a period of several years could be identified from the available research [21, 24] none of which however puts a focus on the MLC2006. Thus, the results from this analysis can fill a gap in available research and contribute to current knowledge for informed policy decisions.

## Methods

A preliminary analysis of registered tonnage and MLC2006 ratification was conducted to determine which MoU to include in the final analysis (Table 1). Based on the results it has been decided to omit the Riyadh MoU as no member country has yet ratified the MLC2006. Furthermore, the Black Sea MoU has not been included due to the minimal tonnage registered in its member countries, leaving seven MoU for the analysis. PSC conducted by the US has not been included as the annual reports published by the US Coast Guard are not comparable to those of the MoUs.

**Table 1:** Overview of the Memoranda of Understanding, including members, MLC2006-ratifications and registered tonnage

Memorandum of Understanding	No. of Members^**a**^	MLC- ratifications total (% of members)^**b**^	Reg. tonnage 2017 (thousands)^**c**^	Ratio of total reg. Tonnage (in %)^**c**^
Paris MoU	27	26 (96.30)	281,042	20.23
Tokyo MoU	20	17 (85.00)	492,595	35.45
Indian Ocean MoU	20	13 (65.00)	20,085	1.45
Caribbean MoU	20	12 (60.00)	90,241	6.49
Abuja MoU	17	9 (52.94)	148,237	10.67
Acuerdo Latino	14	4 (26.67)	227,865	16.40
Mediterranean MoU	10	7 (70.00)	103,352	7.44
Black Sea MoU	6	3 (50.00)	13,840	1.00
Riyadh MoU	6	0 (0.00)	12,223	0.88
			**1,389,480**	

^a^Data on member states from the respective websites of the MoU, extracted in September 2019: Paris MoU to Mediterranean MoU [27–33], Black Sea MoU [34], Riyadh MoU [35]

^b^Data on ratification from The International Labour Organization Database NORMLEX [36]

^c^Data on registered tonnage from the United Nations Conference on Trade and Development UNCTADstat database [37]

All MoU publish freely available annual reports in the publication section of their respective website [27–33], listing data on the inspections carried out, the reported deficiencies and detentions sorted e.g. by ship types, classification society and categories. For this paper, the annual reports from 2010 to 2017 of seven MoU have been viewed and the numbers on annual inspections, deficiencies and detentions and in particular data for deficiencies related to working and living conditions (WLC) and certificates and documents (CD) have been extracted in September 2019. The time frame has been selected to include the time prior to the introduction of the MLC2006 and inspect any potential differences after 2013. Living and working conditions are inspected under the MLC2006 and prior to that the ILO 147. Convention No. 147 is also called the “Merchant Shipping (Minimum Standards) Convention” and has been in force since 1981 [38]. Starting with the entry into force of the MLC2006, some MoUs report separate numbers for these two ILO-Conventions as not all respective member countries have ratified the MLC2006 and some ships were thus still inspected under ILO 147. For the analysis these data have been combined. This allows for a holistic analysis of deficiencies related to WLC across all included MoU. Insights on the attention paid to and compliance with WLC regulations can also be drawn from the combined data. WLC deficiencies cover aspects such as the accommodation, conditions of employment, health protection, medical care and safety (for a more detailed list, see for example the Paris MoU list of deficiency codes [39]). The category CD includes, among others, the audit of the Maritime Labour Certificate, the Declaration of Maritime Labour Compliance but also other important labor-related documents such as the records of daily hours of work and rest or medical certification of seafarers [22].

In total, 11 variables have been used for the analysis: MoU, year, total inspections, total deficiencies, detentions, deficiencies reported under ILO147, deficiencies reported under MLC2006, total deficiencies related to WLC, percentage of deficiencies related to WLC of the total deficiencies, total deficiencies related to CD, percentage of deficiencies related to CD of the total deficiencies. The data presented are total numbers, ratios (in percentage) and mean values. The data analysis and graphical representation have been done using R for Intel Mac OS X 10_14_6 and RStudio 1.2.1335.

## Results

The total number of inspections and deficiencies could be recorded for every year from 2010 to 2017 for all MoU included in the analysis. The Acuerdo Latino and the Mediterranean MoU started to report separate MLC2006-related deficiencies in 2015. The Abuja MoU and the Caribbean MoU were the only two not reporting deficiencies related to ILO147 and MLC2006 separately during the period inspected. The Abuja MoU recorded deficiencies by categories (here: WLC, certificates) for the first time in 2012, thus there are missing values for 2010 and 2011. In total, 344 data values have been evaluated in this analysis.

### Total deficiencies and inspections

**Fig. 1:**
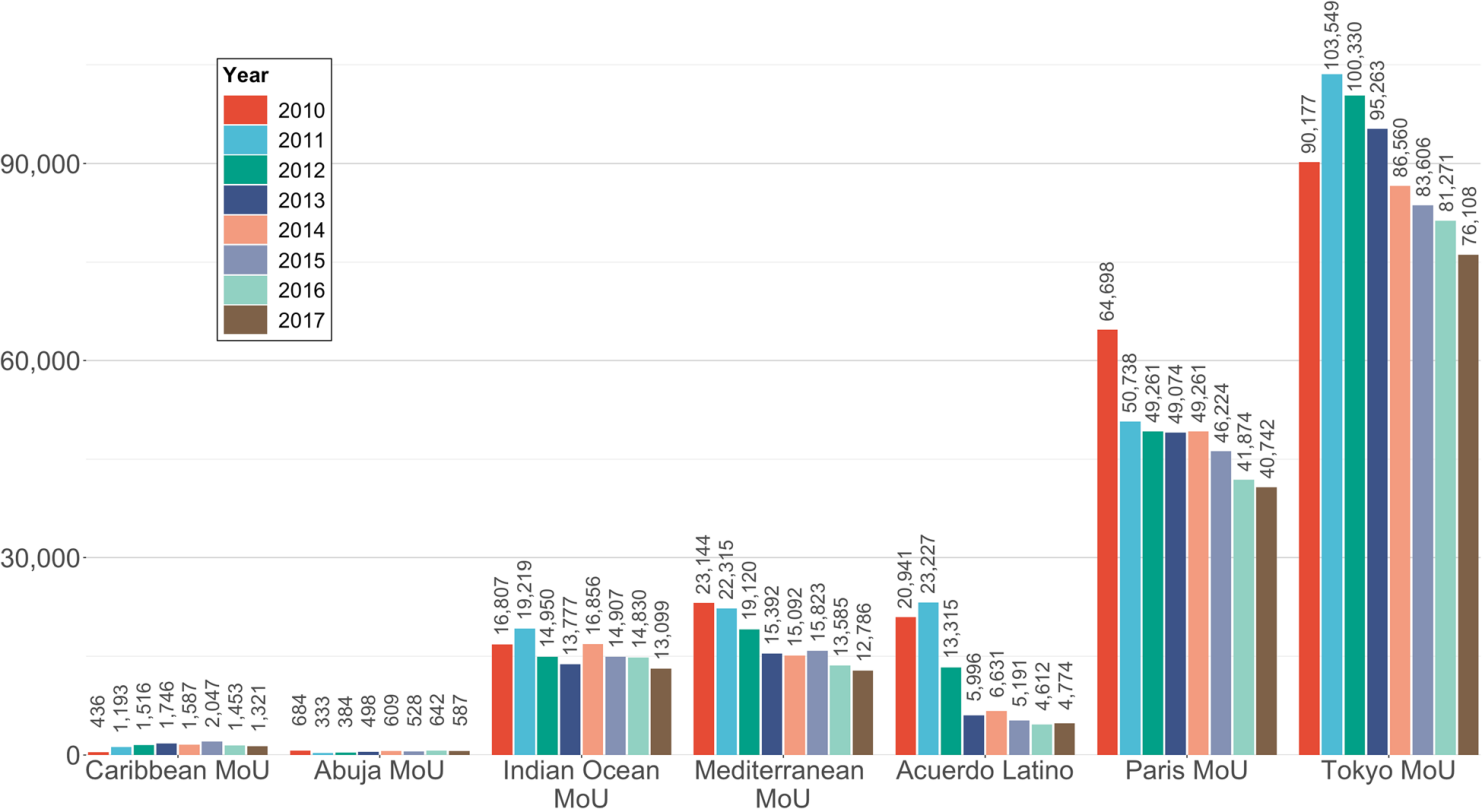
Total number of annually reported deficiencies per MoU between 2010 and 2017

**Fig. 2:**
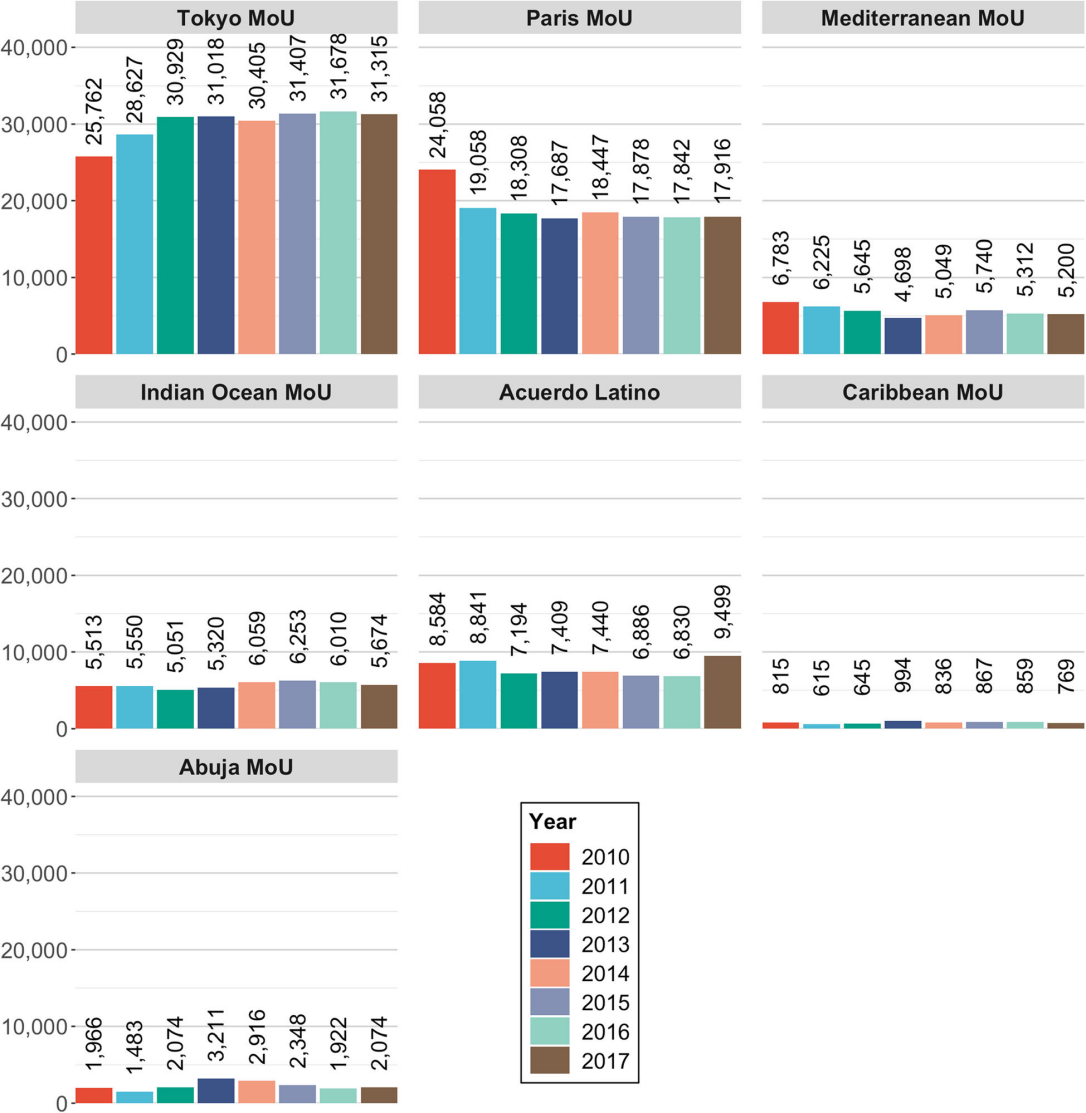
Total number of annually reported inspections per MoU between 2010 and 2017

Figures 1 and 2 show the total numbers per MoU for reported annual deficiencies and inspections respectively. Across all 8 years included in this analysis, the Tokyo MoU was the most active reporting the highest number of inspections and deficiencies despite the Paris MoU having more member states.

Between 2010 and 2017 the overall number of annual deficiencies decreased across most MoU. The decrease was most pronounced for the Acuerdo Latino (− 74.38%), the Mediterranean MoU (− 44.75%), the Paris MoU (− 37.03%), and the Tokyo MoU (− 18.56%). Only the Caribbean MoU recorded an increase from 436 deficiencies in 2010 to 1321 in 2017 (+ 202.98%) (Fig. 1).

The total number of annual inspections remained, on average, at a stable level for most MoU. Thus, the average number of deficiencies found per inspection decreased from 2010 to 2017. The number of inspections conducted by the Tokyo MoU increased from 25,762 in 2010 to 31,315 in 2017, representing a 21.56% increase. The biggest increase was recorded in the the first two years of observation, from 2010 to 2012. After 2012 the number reached a steady level at around 31,000. The Paris MoU reported 24,058 inspections in 2010 before the numbers dropped and stabilized in the following years at around 18,000 inspections. The Acuerdo Latino experienced a drop in the number of annual inspections after 2011 before numbers stabilized from 2012 to 2017 at around 7000 and increased in 2017 to almost 9500 (Fig. 2).

### Deficiencies related to working and living conditions

**Fig. 3:**
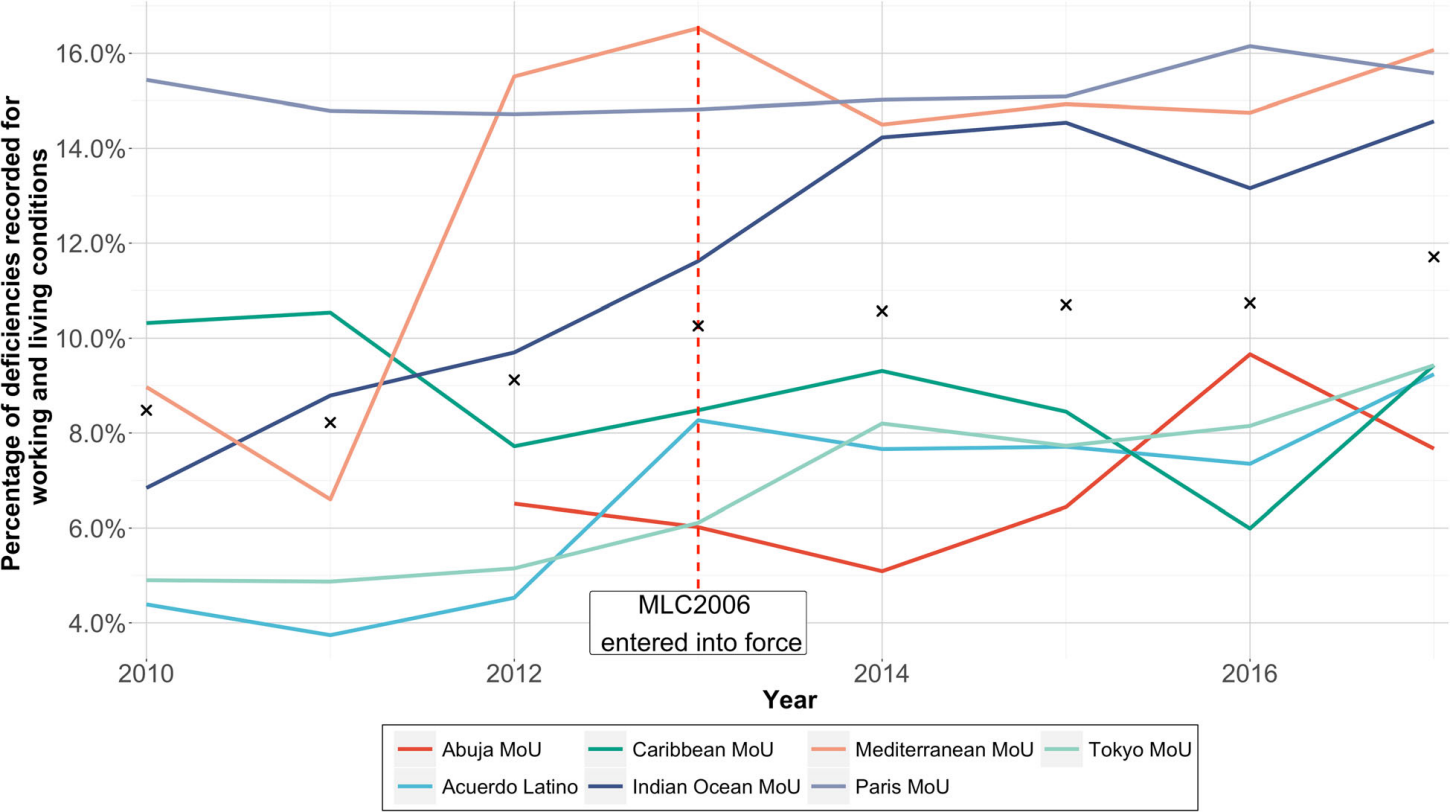
Annually reported deficiencies related to working and living conditions between 2010 and 2017 as percentage of deficiency total (x ≙ annual mean)

The annual number of deficiencies reported for WLC from 2010 to 2017 are visualized as the percentage of the total number of deficiencies in Fig. 3. The annual mean is marked by an x, the vertical dashed line marks the year 2013, i.e. the year the MLC2006 entered into force. There is a large variation among all MoU from 3.74% recorded by the Acuerdo Latino in 2011 to 16.53% recorded by the Mediterranean MoU in 2013. Across all eight years only the Caribbean MoU did not report an increase in WLC deficiencies. The mean ratio of WLC deficiencies increased from 8.48% in 2010 to 11.71% in 2017. After the MLC2006 entered into force, two groups formed, with three MoU reporting ratios above the mean, the other four staying below. The Paris MoU, the Mediterranean MoU and the Indian Ocean MoU recorded consistently higher numbers for WLC-related deficiencies than the group of the other four MoU. However, the year 2017 saw an increase for most of the authorities in the second group as well. The Mediterranean MoU experienced an astonishing increase in the rate of WLC-related deficiencies prior to the MLC2006 from 6.6% (2011) to 15.5% (2012). The Paris MoU, the Caribbean MoU, the Indian Ocean MoU and the Tokyo MoU recorded an increase in the relative amount of deficient WLC after the MLC2006 entered into force. The Abuja MoU, the Acuerdo Latino and the Mediterranean MoU, recorded a decrease but numbers increased again in the following years. The Tokyo MoU, despite reporting the highest number of deficiencies overall, reported a mean of 6.82% of deficient WLC, compared to 15.20% for the Paris MoU and 13.48 and 11.68% for the Mediterranean MoU and the Indian Ocean MoU respectively.

### Deficiencies related to certificates and documents

**Fig. 4:**
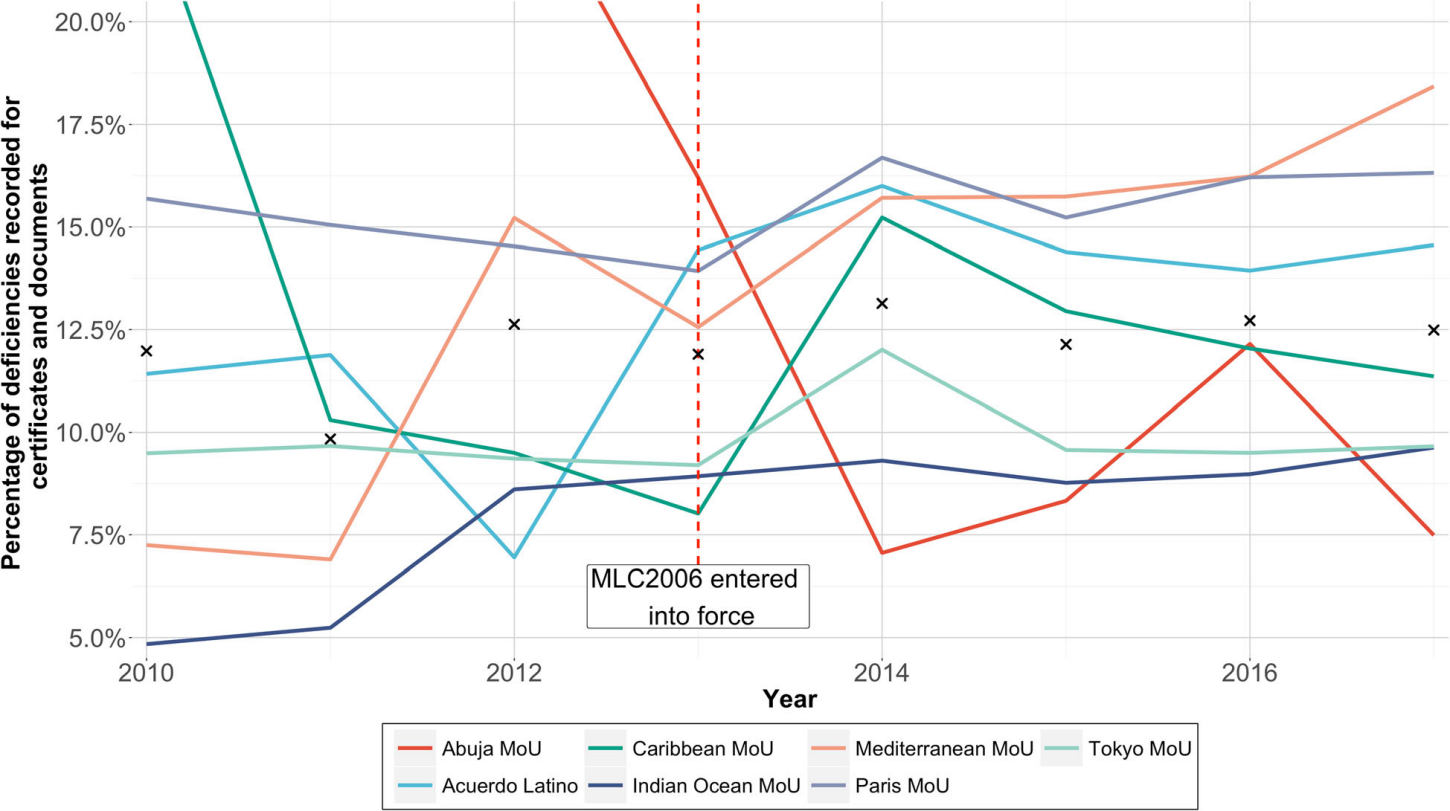
Annually reported deficiencies related to certificates and documents between 2010 and 2017 as percentage of deficiency total (x ≙ annual mean)

Figure 4 shows the number of CD-related deficiencies as the percentage of the toal number of deficiencies. Deficiencies regarding CD make up a higher percentage of the total deficiencies when compared to WLC. With the exception of the Abuja MoU, all other authorities recorded an increase after the MLC2006 entered into force. Overall the mean ratio for CD increased only marginally between 2010 (11.98%) and 2017 (12.49%). There is a large variation between the MoU, but a convergence of values after the MLC2006 entered into force is visible. Three MoU recorded large increases during the period under investigation. The Mediterranean MoU from 2011 to 2012 (+ 8%), the Acuerdo Latino from 2012 to 2013 (+ 8%) and the Caribbean MoU from 2013 to 2014 (+ 9.2%). Numbers for the Abuja MoU, dropped rapidly from 2012 to 2014 by 17.16% followed by an increase and another decrease to the previous level. During the first year of the observed period, the Caribbean MoU recorded a significant drop in issues reported on CD (− 12.87%) but these number increased again after the MLC2013 entered into force.

## Discussion

Six out of seven MoU recorded a decline in deficiencies overall, alongside a relatively stable number of inspections, an indication for a continuous improvement of conditions in the industry and on board in general. Most MoU recorded an increase in the ratio of WLC-related deficiencies, a sign for an increased focus on seafarers’ conditions and compliance with the legal requirements of the MLC2006. The data for deficient WLC evaluated in this paper did not follow the trend hypothesized by the authors. Analyzing the data for certificates and documentation, an argument can be made for an impact of the MLC2006 in some regions. In alignment with the intial assumption, six out of seven MoU reported an increase of deficiencies related to CD in 2014 followed by a decline and thus a potential improvement of conditions in 2015. Only the Abuja MoU recorded data that did not fit the trend predicted by the hypothesis. For the category CD, the mean did not show a significant change in the number of reported deficiencies refuting the assumption of improvements. Overall, the introduction of the MLC2006 did not have the predicted effect on PSC deficiencies recorded for WLC or CD. While the large number of ratifications of MLC2006 show that countries are willing to improve seafarers’ conditions, especially in ships flying flags of non-ratifying countries, other studies also confirm that the MLC2006 did not have the desired effect on board [16, 19].

Several other authors analyzed the impact of the MLC2006 using statistics from PSC inspections [13, 16, 22, 23]. One of the earliest analyses of PSC data related to WLC was conducted by Payoyo [23] using numbers reported by the Paris MoU between 1982 and 1992. The evaluation focused on two major Conventions, one of which was the 1976 Merchant Shipping (Minimum Standards) Convention (ILO 147), the precursor of the MLC2006. The data showed an increase in deficiencies with an increase in inspections. The author concluded that the inspection regime is effective in detecting deficiencies and helps to increase compliance [23]. While it is true that this might indicate an increased attention to WLC, an increase in deficiencies over several years also shows that numerous substandard ships still exist. Almost 30 years later, the present analysis paints a more positive picture with declining deficiencies across a relatively stable number of inspections, thus showing an actual improvement in conditions. An analysis of data from the Swedish Maritime Authority for the period 1996–2001 showed that deficiencies reported during a PSC inspection were reduced by 63% in the next inspection [9]. Although the authors did not focus solely on MLC2006-related statistics, this confirms the trend visible in the numbers collected for this analysis. As the number of deficiencies recorded per inspection is reduced, it suggests a reduction of substandard ships with deficient conditions.

Three authorities (Paris MoU, Mediterranean MoU, Indian Ocean MoU) put an increasing focus on WLC during the time period investigated, while the other four MoU still reported ratios of less than 10% in 2017. The two European MoU, the Paris MoU and the Mediterranean MoU, stand out by also reporting the highest ratios for deficiencies related to CD between 2015 and 2017. This emphasizes the leadership role of European countries in the global shipping industry. Regulations of the European Union (EU) are often more protective, exhaustive and more strictly enforced than those of other countries [40]. European countries have a strong influence on the development of global standards and often take on a pioneer role regarding the ratification of new legislations [16]. The variety between authorities points to the fact that efforts must be taken to harmonize PSC procedures in the future, an issue previously mentioned by other researchers [11, 13, 16, 21]. This would also help to avoid region-shopping, i.e. the practice of ships sailing to ports with weaker PSC inspection routines [12].

Characteristics such as ship type, ship age and equipment influence the likelihood for detection of deficiencies in different categories as shown by Cariou et al. [25] and should be taken into account for efficient targeting of high-risk ships. The calculations showed that when investigating working conditions, a focus on bulk and dry cargo vessels can help to increase the efficiency and effectivity of PSC inspections. While these data could reveal additional information, they are not available for individual conventions but only on an aggregated basis for the whole MoU authority and could not be included in the analysis for this paper. It is also likely that the training of the PSCO, the inspection quality and the behavior of relevant stakeholders such as shipowners can have an influence on PSC statistics and some of the effects seen in this analysis. This is however beyond the scope of this paper.

This study has some limitations that reduce the relibailty of the conlcusions drawn. The data collected and compared was extracted from the annual reports of different MoU authorities which can cause some problems. Each MoU has its own agreement, which is mostly based on that of the Paris MoU but can contain specific requirements and is executed more or less strictly in different regions (see also Supplementary file 1). The policies for targeting and selection of ships to inspect differ. While the Paris MoU has the most sophisticated policies, many of the smaller MoU are not as stringent and several countries are a member of more than one MoU. These aspects impact the inspection regimes and therefore the comparability of the data. Furthermore, not all MoU member states have ratified the same international regulations which hampers harmonization of inspection routines. Lastly, the member states are very heterogeneous regarding their economic development and therefore do not have the same resources available for PSC [12]. This is an observational analysis and the data can only present possible trends. No causality can be determined between the observed increases and decreases in the reported deficiencies and the entry into force of the MLC2006. However, the data collected and presented for this study provide a first overview of current developments and a basis for future research and policy activities. In combination with studies involving both the perspective of seafarers and the industry, a more comprehensive evaluation of MLC2006 could be achieved.

## Conclusion

Seafarers are an essential workforce that guarantee the operation of an important economic pillar in a globalized world. Ensuring their welfare through stringent conventions, such as the MLC2006, is fundamental. PSC is certainly one of the most important instruments for the assurance of adequate conditions in the seafaring industry, in particular for seafarers. Recent years have seen a decrease in reported deficiencies alongside a stable number of inspections, indicating a reduction of substandard ships and an improvement of conditions overall. This analysis could not show a definite impact of the implementation of the MLC2006 on relevant PSC statistics. Several issues remain for seafarers which the MLC2006 fails to adequately address. Statistics such as those from PSC can only present a broad picture of the situation without insight into the details. Data obtained directly from seafarers, ship owners and PSCOs might paint a clearer picture of aspects warranting improvement. Thus, further research in this field and a potential revision of the convention to include these insights are necessary steps for the future. As demonstrated in this paper, there are still large differences among the world’s MoU regarding the prioritization of MLC2006-requirements. Increased collaboration and the harmonization of inspection routines are essential to improve the impact of PSC and thus the conditions and the security in the shipping sector. In the face of the coronavirus (COVID-19) pandemic it is now more important than ever to protect and support seafarers and ensure that they can continue their work safely.

## Supplementary information

**Additional file 1.** Inspection differences among the MoU. More detailed description of the differences in inspection regimes among the MoU including additional literature.

## Data Availability

All data generated or analyzed during in this paper are publicly available in the annual reports on port state control which are published on the respective websites of the MoU.

## Abbreviations

MLC2006: Maritime Labour Convention, 2006
ILO: International Labour Organization
PSC: Port state control
MoU: Memoranda of Understanding
US: United States
PSCO: Port state control officer
WLC: Working and living conditions
CD: Certificates and documents
EU: European Union
