# Supplementary material for: The impact of the Maritime Labor Convention on seafarers’ working and living conditions: an analysis of port state control statistics
Source: BMC Public Health. 2020 Oct 21;20:1586. doi: 10.1186/s12889-020-09682-6 (PMC7576973; doi:10.1186/s12889-020-09682-6)
Supplement: Supplementary file 1 — Additional file 1. Inspection differences among the MoU. More detailed description of the differences in inspection regimes among the MoU including additional literature. [file 12889_2020_9682_MOESM1_ESM.docx]

**Supplementary file 1: Inspection differences among the MoU**

Even though all MoU agreements are based on the original text written for the Paris MoU and are therefore almost identical, differences exist regarding inspection procedures, targeting criteria and institutional agreements. H-S Bang and D-J Jang [1] published a comprehensive overview of all nine MoU and their different legal structure.

The procedures for inspection, ratification and detention as stated in the Paris MoU are the same for all other MoU. However, with the exception of the Tokyo MoU, most other authorities lack the infrastructure, financial capacities and effective policies to conduct equally effective inspections. Organizationally all MoU follow the example of the Acuerdo Latino with a simpler structure than that of the Paris MoU. The Paris MoU and the Tokyo MoU are the most active ones with an inspection target rate of 90% (100% of high-risk ships) for the Paris MoU and 80% for the Tokyo MoU. Other MoU have lower targets with 20% for the Acuerdo Latino, 15% for the Caribbean MoU, the Abuja MoU and the Black Sea MoU and 10% for the others. Targeting criteria differ and only the Tokyo MoU has defined requirements as sophisticated as those of the Paris MoU. Often, not all member states have ratified the same international conventions which further hampers harmonization of PSC procedures. All in all, industry experts agree on the fact that the differences among MoU inspections are still too large and must be further harmonized [1-3].

1. Bang H-S, Jang D-J. Recent developments in regional memorandums of understanding on Port State Control. Ocean Dev. Int. Law. 2012; doi:10.1080/00908320.2012.672293.

2. Knapp S, Van de Velden M. Visualization of differences in treatment of safety inspections across Port State Control regimes: a case for increased harmonization efforts. Transport Reviews. 2009; doi:10.1080/01441640802573749.

3. Cariou P, Mejia MQ, Wolff F-C. Evidence on target factors used for port state control inspections. Mar. Policy. 2009; doi:10.1016/j.marpol.2009.03.004.
